# Fitting the epidemiology and neuropathology of the early stages of Alzheimer’s disease to prevent dementia

**DOI:** 10.1186/s13195-014-0079-9

**Published:** 2015-01-15

**Authors:** Javier Mar, Myriam Soto-Gordoa, Arantzazu Arrospide, Fermín Moreno-Izco, Pablo Martínez-Lage

**Affiliations:** Clinical Management Unit, Alto Deba Hospital, Avenida Navarra 16, Mondragon, 20500 Spain; Health Services Research on Chronic Patients Network (REDISSEC), Avenida Navarra 16, Mondragon, 20500 Spain; AP-OSI Research Unit, Alto Deba Hospital, Avenida Navarra 16, Mondragon, 20500 Spain; Department of Neurology, Donostia Hospital, C/ Dr Beguiristain s/n, Donostia-San Sebastián, 20014 Spain; Fundación CITA-Alzheimer Fundazioa, Pº Mikeletegi 71, Donostia-San Sebastián, 20009 Spain

## Abstract

**Introduction:**

Recent research on biomarkers has made possible the diagnosis of pre-dementia and even preclinical Alzheimer’s disease (AD), thus providing the ideal context for prevention. The aim of this study was to investigate the epidemiology of the early stages of AD by fitting neuropathologic and epidemiological data to assess the feasibility of prevention programs.

**Methods:**

The study addressed primarily the construction of a discrete event simulation model of the stages of dementia. Age was included in the mathematical functions to combine the two competitive risks that determine the epidemiology of AD, that is, time to onset of dementia and time until death by other causes. Subsequently, this model was calibrated to reproduce the prevalence of pathological findings associated with AD. The beginning of the preclinical stage was taken to coincide with Thal phase 1 deposition of amyloid-beta. The duration of the prodromal stage, marked by mild cognitive impairment, was based on a 10% annual conversion rate from this level of impairment to dementia. The validation of prevalence figures also permitted estimation of the incidence and duration of preclinical and prodromal stages.

**Results:**

In Spain, half of the nearly 10 million people aged more than 60 years are in the early stages of AD; 35.9% are in a preclinical stage, and up to 14.2% are in a prodromal stage. However, dementia will develop in only 38% of this population. The weighted mean time to dementia was 22.0 years from the start of Thal phase 1 and 9.0 years from the start of phase 2. Results of simulation models showed a lack of correlation between clinical and pathological classifications.

**Conclusions:**

These findings raise questions about the feasibility of drug-based prevention strategies. Currently, screening programs with biomarkers in the early stages of AD cannot be applied to the half of the general population older than 60 years. Hence, intensive research is needed regarding risk factors, so that more affordable strategies may be planned. More efficient criteria are also needed to select those subjects with mild cognitive impairment who have an increased probability of positive screening for biomarkers (prodromal stage).

**Electronic supplementary material:**

The online version of this article (doi:10.1186/s13195-014-0079-9) contains supplementary material, which is available to authorized users.

## Introduction

The definition of Alzheimer’s pathology and Alzheimer’s disease (AD) has been the subject of profound conceptualization [1]. The research diagnostic criteria proposed by the National Institute of Neurological and Communicative Disorders and Stroke and the Alzheimer’s Disease and Related Disorders Association Work Group in 1984 characterized AD as a type of dementia in which the clinical diagnosis could be determined on an exclusionary basis and confirmed only post mortem [2]. For more than 25 years, this approach was generally embraced, until advances in biomarker research reached the clinical setting and promoted a new paradigm [3]. The International Work Group [4] and the National Institute of Aging–Alzheimer’s Association task force [5,6] proposed new diagnostic criteria that cover all possible clinical manifestations of the disease and allow a premortem biological diagnosis to be made on the basis of positive biomarkers. Moreover, the concept of a preclinical stage of AD, with no cognitive or behavioral symptoms, has been defined as the finding of positive biomarkers of amyloid deposition with or without neurodegenerative changes [7]. Accordingly, AD is defined as a long, degenerative process that starts with the development of specific neuropathological changes in the brain without clinical manifestations (preclinical stage) until progression to a prodromal stage of mild cognitive impairment (MCI) and finally to dementia [1]. Available empirical information about the early preclinical stage comes mainly from brain registries [8], although *in vivo* information is now being gathered with biomarker studies [9].

The fact that AD may now be detected in its earliest symptomatic, or even in its asymptomatic, preclinical stage has opened new appealing lines of research to investigate potential prevention strategies at the secondary or tertiary level. Prevention strategies could reduce the population burden of AD through the introduction of disease-modifying drug treatments or intervention programs for risk-factor modification [10,11]. However, the targets and time periods of both observational and interventional studies are too limited to estimate the long-term impact of prevention at the general population level, and many questions regarding feasibility remain. The reproduction of the natural history of AD with mathematical models has been used to predict its evolution through simulation and to evaluate the health impact and cost-effectiveness of intervention programs [12,13]. In addition, such models may help in calculating epidemiological parameters of prevalence, incidence and duration of disease stage [14].

The objective of this study was to estimate the epidemiology of early stages of AD by fitting the incidence and prevalence with neuropathological findings of AD in autopsies in the general population to assess the feasibility of prevention programs.

## Methods

The study primarily addressed the construction of a validated model of the dementia stages on the basis of the results of two meta-analyses that estimated the incidence and prevalence of AD dementia in European populations [15,16]. Subsequently, this model was calibrated to reproduce the prevalence of pathological findings associated with AD in the population by age group, according to the study by Braak and colleagues [8]. The epidemiology of the early stages of AD was described by calculating the incidence, prevalence and duration of the preclinical and prodromal stages. As there is currently no empirical data to estimate these parameters in any population, a discrete event simulation (DES) model was used to represent the relation of AD to the Spanish population more than 40 years old and to calculate them [14]. The Technical Appendix (Additional file 1) contains the complete description of the model and its parameters. Discrete event simulation is a flexible modeling method characterized by the ability to represent complex behavior within, and interactions between, individuals, populations and their environments [17]. This study was based on a literature review and a computing model without patient involvement, so no ethical approval or consent was needed.

### Conceptual model

The natural history model divides AD into three clinical stages: preclinical, prodromal and dementia [4-6]. Because the clinical classification of AD does not correlate with the presence and density of deposits of amyloid beta (Aβ) and tau proteins in the brain [18], the discrete event simulation model was populated directly with findings associated with progress of Aβ deposition [8]. Clinicopathological studies indicate that the presence of Aβ is more specific than tau deposits for the etiology of AD [18,19], so we reproduced in the model the evolution of neuropathological findings by Thal phases [8,20]. Braak and colleagues analyzed 2,332 brains for findings associated with AD and estimated the presence of Aβ deposits in terms of the Thal phases by age group [8]. From these results, we identified the beginning of the preclinical stage of AD by Thal phase 1, which is characterized by the presence of plaque-like Aβ deposits in the temporal neocortex. The next stages showed deposits also in the allocortex and associated areas of the neocortex (Thal phases 2 and 3) or in virtually all cerebrocortical regions (Thal phase 4) [8,20]. In the representation of the conceptual model of AD, the relevant input was only the time from the beginning of the Thal 1 phase. The progression of pathological findings according to Thal phases was calculated to compare the results with estimates based on clinical classification.

Beyond Aβ deposition, the duration of the preclinical stage depends on different factors that still remain to be discovered. The combination of these conditions and the competitive risk of death from other causes determines which individuals reach the clinical stage. As the National Institutes of Health State-of-the-Science Conference noted, there is currently no evidence about modifiable factors causally associated with AD [21]. However, Barnes and Yaffe identified factors consistent with evidence of AD, such as diabetes mellitus, present smoking, depression, cognitive impairment, physical activity and poor diet (high saturated fat and low vegetable intake) [11]. Following their approach in our study, we used the term risk factors instead of risk markers to account not only for the current scenario but also for potential future findings; however, we stress that this use does not indicate any demonstrated causal relationship between epidemiological condition or behavior and AD.

Given the lack of correlation of prodromal stage with Thal phases, the duration was estimated from studies measuring the conversion of MCI to dementia with rates that ranged from 5 to 20% per year [18,22]. This variability derives from the heterogeneity of samples from patients with MCI, which includes both clinical studies (high rates) and population studies (low rates), so we applied a 10% rate that resulted in a duration of 10 years by application of the exponential function [23].

### Discrete event simulation model

**Figure 1:**
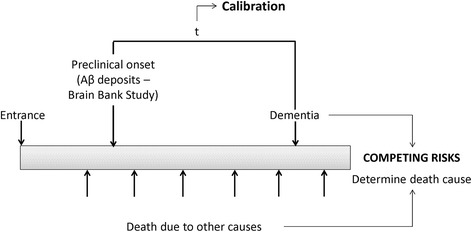
**The natural history of Alzheimer’s disease by clinical and pathological classifications.** Aβ, amyloid beta.

The model was preloaded with the entire Spanish population older than 40 years of age in 2009 to measure the stage of AD in individuals in 2010. On entry into the simulation model, individuals were characterized by the relevant attributes of age, sex, duration until death and duration until dementia (Figure 1). The stochastic nature of the process meant that the individual’s behavior was randomly determined in the model, depending on which risk (either the development of dementia or death by other causes) finally occurred [14,17]. Table 1 presents the main parameters of the model.

**Table 1:** **Parameters of the model**

	**Source**	**Function**	**Parameters**	**Male**	**Female**
Time until death due to other causes	INE	Gompertz	ln(α)	−10.022	−11.922
			β	0.090	0.108
Time until dementia	[15]	Gompertz	ln(α)	−17.825	−16.772
			β	0.164	0.157
Dementia survival	[21]	Polynomic	b0	30.835	42.767
			b1	−0.447	−0.709
			b2	0.002	0.003
Prodromal stage length	[17,18]	Exponential	λ	10	10

INE, Spanish National Institute of Statistics.

We calculated the parameters for the Gompertz function that reproduces life expectancy by age and sex from mortality rates in the Spanish population in 2010, as obtained from the National Institute of Statistics (INE) [24]. It was assumed that during the prodromal and preclinical stages the mortality rate was the same as that in the general population. The same procedure, but with the incidence rates of AD dementia, allowed us to calculate the parameters for the Gompertz function that set the age of onset of dementia [15,24]. The calculated incidence [15] matched the model used by Brookmeyer and colleagues and the results of Ferri and colleagues [12,25]. Dementia incidence was not a model input, but the result of the interaction between two competing risks (death or development of dementia) in cohorts of individuals entering the preclinical stage in the model and was used as a main result of external validation. The prevalence of dementia was the result of the combination of incidence and disease duration, and this was also measured to validate the model. The figures used to parameterize dementia risk function derive from studies that may not be representative enough for ages older than 90 years. Our model could overestimate the weight of such a population, as risk grows over time and, with use of the Gompertz function, reaches high levels for older people. However, this limitation would not apply to the early stages of AD, which are the main concern of this work. Survival after a diagnosis of dementia was determined from the study by Dodge and colleagues [26].

Once we determined the age of onset of dementia, it was possible to identify the starting points of different Thal phases by calibration. For each phase, we established the time from the beginning of Aβ deposition to dementia. The results were obtained in 2010 and grouped by age and sex. Calibration is the process of determining the values of unobservable parameters by constraining model output to replicate observed data [27]. The calibration step consisted of estimating the duration of the preclinical stage by adjusting the model until it matched the observed pattern for Thal phase 1 in the study by Braak and colleagues [8]. Different values were tested by calibration [27], as Thal phase 1 prevalence is a directly unobservable parameter. Epidemiological parameters for Thal phases 2 and 3 were also calculated by applying the same method.

## Results

The model was validated by comparing the calculated life expectancy, incidence and prevalence of the dementia stage in 2010 with the values of Dodge and colleagues, of Fratiglioni and colleagues and of Lobo and colleagues (see Additional file 1) [15,16,26]. The number of individuals with dementia due to AD obtained with the model was 370,300 in 2010, which appears to correlate with the 400,000 people estimated in a review of Spanish surveys by de Pedro-Cuesta and colleagues [28].

The duration to dementia from the start of the Thal phases was variable, with a weighted mean of 22.0 years for phase 1 and 9.0 years for phase 2 (Table 2). Table 3 presents the disaggregated incidence by age group in the Spanish population according to clinical and pathological classifications. The disaggregated prevalence figures by epidemiological and pathological classifications (Table 4) highlight the huge range of early stages of AD in the population. From the population in Spain of nearly 10 million people older than 60 years of age, 35.9% were shown to be in a preclinical stage and 14.2% in a prodromal stage.

**Table 2:** **Time from the start of each Thal phase to the onset of dementia**

	**Time until dementia from**	
**Age**	**Thal1**	**Thal2**
40 to 44	45.6	22.0
45 to 49	35.3	22.0
50 to 54	31.8	21.9
55 to 59	29.8	20.5
60 to 64	28.1	18.5
65 to 69	26.6	16.7
70 to 74	23.1	13.0
75 to 79	19.0	8.7
80 to 84	14.9	4.3
85 to 89	10.8	2.1
90 to 94	6.7	1.0
95 to 99	2.9	0.9
>100	2.1	1.0
Weighted mean	22.0	9.0

**Table 3:** **Incidence of new cases of Alzheimer’s disease by 1,000 person-years and by clinical and pathological classifications**

	**Incidence/1,000 person-years**					
	**Clinical classification**			**Pathological classification**		
**Age group**	**Preclinical**	**Prodromal**	**Dementia**	**Thal1**	**Thal2**	**Thal3**
40 to 44	24.01	0.13	0.00	23.07	1.68	1.11
45 to 49	5.61	0.31	0.00	5.61	2.58	0.68
50 to 54	7.53	0.64	0.16	7.53	4.98	1.54
55 to 59	18.51	1.45	0.46	18.51	10.00	2.44
60 to 64	27.60	3.49	0.70	27.60	16.98	3.32
65 to 69	32.76	7.65	1.87	32.76	18.13	7.08
70 to 74	10.43	16.24	4.22	10.43	28.14	12.14
75 to 79	10.34	32.65	7.50	10.34	28.72	14.21
80 to 84	10.10	57.29	15.27	10.10	10.51	20.27
85 to 89	13.80	16.98	35.33	13.80	11.22	26.84
90 to 94	12.09	22.32	61.83	12.09	12.09	36.26
>95	33.85	23.44	109.38	33.85	49.48	104.17

**Table 4:** **Prevalence by age group and type of classification (clinical and pathological) in the Spanish population**

		**Clinical classification** ^**a**^			**Pathological classification** ^**b**^		
**Age group**	**Population**	**Preclinical**	**Prodromal**	**Dementia**	**Thal1**	**Thal2**	**Thal3**
**Number**							
40 to 44	2,981,900	252,700	800	0	253,500	16,900	9,400
45 to 49	3,530,900	388,400	4,900	0	393,300	47,200	19,800
50 to 54	3,109,400	431,800	9,400	1,100	442,300	93,600	31,600
55 to 59	2,620,100	517,600	19,400	3,200	540,200	172,400	49,900
60 to 64	2,438,200	739,200	43,500	9,200	791,900	336,900	82,400
65 to 69	2,091,000	929,800	79,800	17,800	1,027,400	461,000	120,900
70 to 74	1,705,800	817,900	148,900	31,300	998,100	583,200	178,600
75 to 79	1,653,700	699,100	291,800	58,200	1,049,100	814,400	266,400
80 to 84	1,218,300	329,500	418,600	90,500	838,600	709,500	277,300
85 to 89	659,500	67,200	330,800	90,400	488,400	416,600	195,600
90 to 94	215,100	14,100	97,300	52,200	163,600	136,000	74,700
>95	38,400	2,700	9,100	16,400	28,200	22,800	14,100
Total >40	22,262,300	5,190,000	1,454,300	370,300	7,014,600	3,810,500	1,320,700
Total >60	10,020,000	3,599,500	1,419,800	366,000	5,385,300	3,480,400	1,210,000
**Percentage**							
40 to 44	100.0	8.5	0.0	0.0	8.5	0.6	0.3
45 to 49	100.0	11.0	0.1	0.0	11.1	1.3	0.6
50 to 54	100.0	13.9	0.3	0.0	14.2	3.0	1.0
55 to 59	100.0	19.8	0.7	0.1	20.6	6.6	1.9
60 to 64	100.0	30.3	1.8	0.4	32.5	13.8	3.4
65 to 69	100.0	44.5	3.8	0.9	49.1	22.0	5.8
70 to 74	100.0	47.9	8.7	1.8	58.5	34.2	10.5
75 to 79	100.0	42.3	17.6	3.5	63.4	49.2	16.1
80 to 84	100.0	27.0	34.4	7.4	68.8	58.2	22.8
85 to 89	100.0	10.2	50.2	13.7	74.1	63.2	29.7
90 to 94	100.0	6.6	45.2	24.3	76.1	63.2	34.7
>95	100.0	7.0	23.7	42.7	73.4	59.4	36.7
Total >40	100.0	23.3	6.5	1.7	31.5	17.1	5.9
Total >60	100.0	35.9	14.2	3.7	53.7	34.7	12.1

^a^Data for clinical stages are not aggregated. ^b^Data for pathological classification prevalence are aggregated.

**Figure 2:**
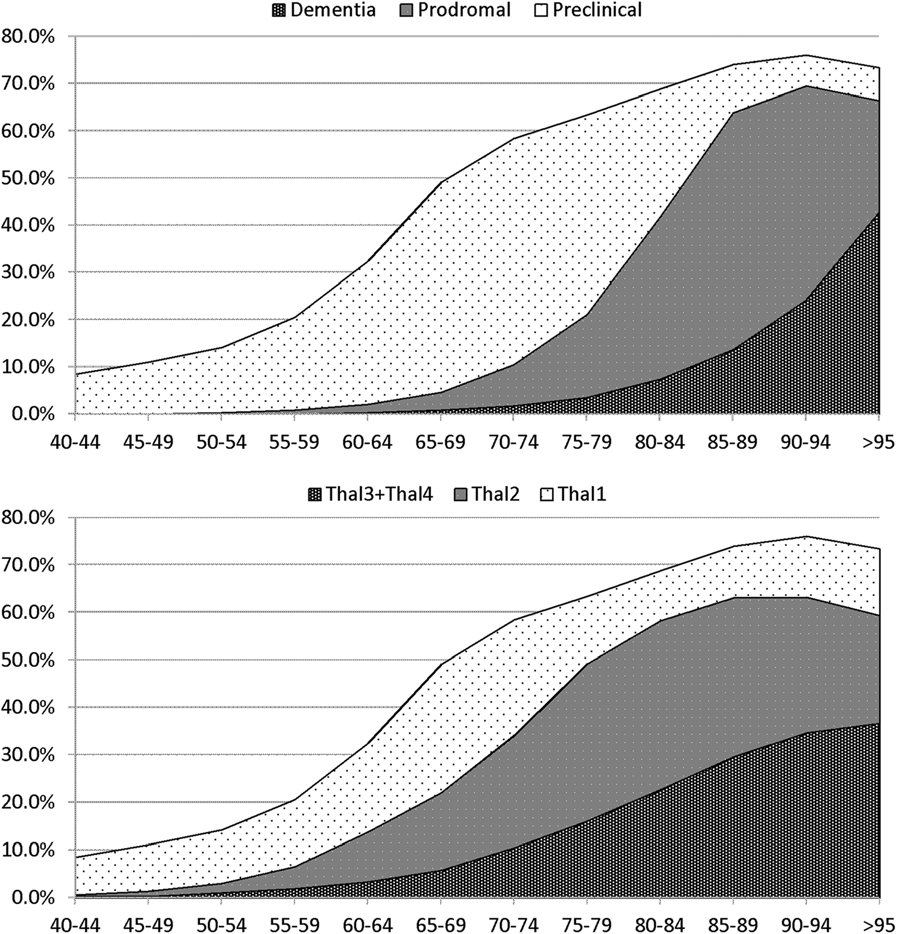
**Disaggregated prevalence by age group and by clinical and pathological classification.**

Figure 2 shows the percentage of individuals in each age group according to clinical and pathological classifications. The same data, but incorporating the figures of Braak et al. for Thal phases, appear in the Technical Annex to display the goodness of fit for results (see Additional file 1).

## Discussion

One of the main findings of this research is that one-half of the population older than 60 years of age in a developed country has some degree of Aβ deposition in the brain. This result agrees with those from population-based autopsy studies, such as the Medical Research Council Cognitive Function and Aging Study that showed 47% of nondemented subjects older than 70 years had mild, moderate or severe densities of neuritic plaques [29]. The enormous size of such a group of affected individuals is highlighted by the fact that it would encompass 15.2% of the total Spanish population (46,148,700 persons). Since the lifetime risk of AD at age 60 years is 19%, an intervention program targeting the 50% of subjects with Aβ deposits would include treating 68% of that population who would never reach the dementia stage [30]. With use of both neuropathological and clinicoepidemiological data, discrete event simulation modeling has provided an estimate of the time lapse between the earliest preclinical changes and the onset of dementia. This interval is usually considered to range from 10 to 20 years [5], but according to the model may be more than 30 years. The findings of both high prevalence and prolonged asymptomatic periods have important public health implications in the study of dementia, particularly in the prevention of AD. Salomon and colleagues have discussed the relevance for prevention plans based on two different concepts of AD onset [30]; that is, whether the starting point relies on pathological changes only [5-7] or on specific clinical manifestations [4]. Consistent with the definition of AD onset, the same preventive intervention can be labeled as primary when dealing with clinical symptoms only or as secondary if prior pathological changes are considered. In relation to our use of Aβ deposition in the model as the marker of the preclinical stage, we understand that primary prevention interventions should be applied before the onset of pathological changes by promoting initiation and maintenance of good health and eliminating or modifying potential risk conditions, if these are ever ascertained [30,31]. We believe secondary prevention would target subjects at the preclinical phase to avoid progression to overt clinical AD and this objective could be hypothetically achieved by risk-factor intervention or disease-modifying therapies. In turn, interventions in the prodromal stage would qualify as tertiary prevention to target the delay or avoidance of dementia onset in the presence of symptoms. In keeping with these definitions and our results, the size of the population targets would include the whole nondemented population for primary prevention, 23.3% of that population older than 40 years for secondary prevention, and 14.2% older than 60 years for tertiary programs. The size of these target populations constrains the viability of an ideal public health perspective to reduce AD prevalence based on a scalable approach, with interventions first targeting risk factors and then pharmacologic treatment in a highly selected group of individuals [9].

Much information has been gathered regarding the role of vascular risk factors and certain lifestyle conditions, such as low education, diet or sedentary behavior, and their association with dementia in general and AD in particular [32]. However, at present, no causal relationship has been demonstrated, and there is no evidence that the reduction of risk factors directly results in decreased incidence of either biomarker positivity or clinical AD [11]. Prevention strategies with anti-amyloid therapies have been proposed for asymptomatic individuals with the genetic and sporadic forms of AD [33]. However, our findings suggest that pharmacologic interventions in subjects with preclinical sporadic AD are hardly feasible. First, screening programs to detect amyloid based on positron emission tomography are so far unaffordable, and those based on lumbar puncture are invasive and need further standardization. Second, even if a more accessible biomarker becomes available and a therapy shows sufficient efficacy and safety, its application would target the 50% of subjects older than 60 years who have amyloid deposits, 62% of whom would never reach the dementia stage [34]. Overdiagnosis is a concept borrowed from cancer-screening evaluation that can also be applied to AD research; in this situation, overdiagnosis describes those individuals found to have AD by the presence of positive biomarkers but who will never have symptoms [35]. The risk of falling into the big prostate mistake has already been highlighted [36].

Tertiary programs might hypothetically treat symptomatic subjects with MCI due to AD to delay or prevent progression to the dementia stage. According to our data, it is still difficult to take a different approach for people with MCI. Subjects with MCI are at a significantly increased risk for the development of dementia associated with AD [37], and therefore represent a target population for preventive strategies. In our model, if the intervention in 2010 had been limited to subjects older than 60 years of age in the prodromal stage (MCI with positive AD biomarkers), the size of the target population would have been 14.2% (1,419,000 individuals). However, to make the diagnosis in this population, subjects with general MCI or MCI of any type should be screened with the appropriate AD markers, but they are not yet widely available. The size of the population with general MCI limits the generalized use of biomarkers or therapies and shows the need for more specificity in identification of the target population. Identifying those subjects with MCI who have a high probability of biomarker positivity would help to select those at high risk of rapid conversion to dementia. Improved predictive values at early diagnosis would result in fewer treated individuals who otherwise would never have had dementia. It is true that this approach can be only speculative before a disease-modifying treatment is available, but subjects with MCI and a positive biomarker could well be targeted in clinical trials with the already approved, cheaper cholinesterase inhibitors in an attempt to delay onset of dementia [38]. The definition of MCI from clinical studies has determined the super-selection of patient groups at high risk of dementia [39], but direct application of such data to population-based programs may not be completely appropriate. On one hand, the clinical course of MCI is not always progressive. On the other, not all subjects in whom dementia develops have previously fulfilled the diagnostic criteria for MCI [39].

One of the strengths of our work is the combination of the two competitive risks that determine the epidemiology of AD: time to onset of dementia and time until death by other causes. The inclusion of age in the mathematical functions and the thorough validation process (see Additional file 1) shows that our AD model has not ignored the ‘elephant in the room’ analogy described by Brayne in her seminal paper [40]. Our approach to AD is a view extending over the patients’ lifetime and incorporating the long preclinical stage that can only be characterized by progressive neuropathological changes. We also acknowledge some limitations in our approach. First, modeling findings should always be interpreted with caution, because they are based on a mathematical representation of the disease and not directly on empirical data. However, our representation of the natural history of AD is robust because it relies on an integrative framework that includes clinical, neuropathological, demographic and epidemiological elements. Second, we did not address the current multifactorial expression of the course of AD in late life in terms of cognitive impairment and dementia [31]. Defining mathematical functions to explore the role of vascular factors or other comorbidities in the development of the early stages of AD should be the object of further analysis. As an initial approach, we have reproduced a pure AD model based on Aβ deposition. However, we acknowledge that understanding the role of vascular and lifestyle-related factors is a key issue in the research agenda for dementia prevention and including them will be an advance in the mathematical representation of the natural history of AD. Third, estimating the prevalence of the preclinical and clinical stages from a model grounded in neuropathological data confronts the problem of the well-established lack of correlation between amyloid neuropathological findings and clinical manifestations [18,19]. However, our model is intended to provide epidemiological indicators to characterize the time from the onset of AD-related changes in the brain until the onset of clinical milestones, not to support or question the pathogenic or bystander role of amyloid. Simulation models can integrate existing partial knowledge of preclinical and prodromal phases of AD in joint representation of its natural history and assessment of the impact of possible interventions in terms of health benefit and economic burden. As has been noted in other studies with different approaches, results of simulation models show the lack of correlation between clinical and pathological classifications [18,19]. Aβ (Thal phase 1) is present in a significant percentage of the population 65 to 70 years of age; it seems to increase thereafter and plateau in the population aged 85 years or older. These differences support the hypothesis that Aβ accumulation is necessary, but insufficient alone, to cause dementia; other factors including vascular conditions, inflammation, brain reserve and cognitive reserve are also involved.

## Conclusions

These findings raise questions about the feasibility of drug-based prevention strategies. Currently, screening programs with biomarkers in the early stages of AD cannot be applied to the one-half of the general population older than 60 years of age. Intensive research regarding risk factors is therefore needed, so that more affordable strategies may be planned. More efficient criteria are also needed to select those subjects with MCI who have an increased probability of positive screening for biomarkers (prodromal stage).

## Additional file

Additional file 1:
**Presents the Technical Appendix for the AD model.**

## Abbreviations

AD: Alzheimer’s disease
Aβ: amyloid beta
MCI: mild cognitive impairment
