# Supplementary material for: Fitting the epidemiology and neuropathology of the early stages of Alzheimer’s disease to prevent dementia
Source: Alzheimers Res Ther. 2015 Jan 15;7(1):2. doi: 10.1186/s13195-014-0079-9 (PMC4338563; doi:10.1186/s13195-014-0079-9)
Supplement: Additional file 1: — Presents the Technical Appendix for the AD model. [file 13195_2014_79_MOESM1_ESM.pdf]

Title: Fitting the epidemiology and neuropathology of the early stages of Alzheimer's disease to prevent dementia.

Short title: Fitting the epidemiology and neuropathology of Alzheimer's disease.

Authors: Javier Mar 1,3, Myriam Soto-Gordoa 2,3, Arantzazu Arrospide 2,3, Fermín Moreno-Izco 4, Pablo Martínez-Lage 5.

Affiliations.

1 Clinical Management Unit. Alto Deba Hospital. Mondragon. Spain.

2 AP-OSI Research Unit. Alto Deba Hospital. Mondragon. Spain.

3 Health Services Research on Chronic Patients Network (REDISSEC).

4 Department of Neurology. Donostia Hospital. Donostia-San Sebastián. Spain.

5 Fundación CITA-Alzheimer Fundazioa. Donostia-San Sebastián. Spain.

TECHNICAL APPENDIX OF ALZHEIMER'S DISEASE MODEL

INTRODUCTION

Mathematical models that represent the natural history of a disease are complex systems that combine inputs from different sources to achieve a research result. As this characteristic could hinder their credibility, a full description of such models is required to explain their components in detail and to avoid the black box effect. This technical appendix is intended to present the details of the model to make it transparent and understandable.

CONCEPTUAL MODEL

The objective of this study was to estimate the epidemiology of early stages of Alzheimer's disease (AD) by fitting their incidence and prevalence with neuropathological findings associated with AD in general population autopsies to assess the feasibility of prevention programs. Therefore, the model must represent not only clinical but also pathological factors.

The clinical representation of AD was based on the recommendations published by the National Institute on Aging and Alzheimer's Association which divide AD into three stages: preclinical, prodromal, and clinical. The pathological classification of beta-amyloid (Aβ) deposits was made according to the classification of Thal. As this classification is not well correlated with dementia, we decided to represent only its first three phases [1].

The conceptual model was, therefore, that outlined in Figure 1 in which entities moved from one state of health to another depending on the initially assigned chronology.

MATHEMATICAL MODEL

The mathematical tool applied to represent the natural history of AD was a discrete event simulation (DES) [2,3]. We used Arena simulation software (version 14; Rockwell Corporation) to program the model.

The model represented the population over 40 years of age, since the prevalence of among younger people is not representative. Once the model was filled with entities (warming-up period), 1)

characteristics that determine the start of the consecutive stages were assigned, and 2) the start of the different stages were allocated and, then, the incidence and prevalence of each stage were measured (Figure 1).

Warm-up

The warming-up period is the time that it takes to fill the model with entities. This time is needed because unlike in the model, in reality, there are prevalent cohorts at the starting point. In this case, the warming-up period was around 60 years since there were individuals with AD who were around 100 years old in the year of analysis. In any case, as the annual population structure is known, the whole population over 40 years of age in 2009 entered in the first year of the model, each of these individuals being assigned with a corresponding health status. The following year only the 40-year-old cohort was added. This way not only prevalence but also incidence could be explored.

Assignment of characteristics or attributes that determine stage starting points

Attributes are the characteristics that define the chronology of events. In this model, in addition to age and sex, the following features were assigned:

- Time to AD dementia
- Time from the beginning of each of the phases/stages (preclinical, prodromal, Thal 1, Tahl 2, Tahl 3) to AD dementia
- Time to death due to AD
- Time to death due to other causes

This information helped to determine the beginning of each stage/phase. At this point, it is important to note that the reference age considered to determine the age of onset of AD is 50 years; this way some individuals would have developed the disease by the age of 60. In contrast, the reference for the age of death from other causes was the age when entities entered in the system, since it was known that individuals were alive at that moment. At the moment when they entered the system, most individuals were in a healthy state, but in some cases Aβ deposition had begun.

Let us analyze as an example what would happen to one of the individuals who entered the system at 60 years of age, if the results of the functions introduced give the following results:

- Time to AD dementia (T_AD) = 35 years
- Time from the beginning of Aβ deposition until AD-dementia onset (T_BA) = 26 years
- Time from the beginning of MCI until AD-dementia onset (T_MCI-Dem) = 5 years
- Time from the beginning of Thal phase 2 until AD-dementia onset (T_Tahl2-Dem)= 22 years
- Time from the beginning of Thal phase 3 until AD-dementia onset (T_Tahl3-Dem)= 11 years
- Time until death due to dementia (T_MEA) = 3 years
- Time to death due to AD (T_DAD) = 3 years
- Time to death due to other causes (T_DOC) = 6 years

From this data, we calculated the start of each of the phases/stages, each in turn allowing us to determine the following one.

- Age at AD-dementia onset (A_AD) = Reference age (50) + T_AD= 50+35 = 85 years
- Age at the beginning of Aβ deposit BA= A_AD-T_BA = 85-26=59 years
- Age at the beginning of preclinical stage = Age at the beginning of Aβ deposit =59 years
- Age at the beginning of prodromal stage = A_AD - T_MCI-Dem = 85-5 = 80 years
- Age at the beginning of Thal 1 phase = Age at the beginning of Aβ deposit =59 years
- Age at the beginning of Thal 2 phase = A_AD- T_Thal2-Dem = 85-22 = 63 years
- Age at the beginning of Thal 3 phase = A_AD- T_Tahl3-Dem = 85-11 = 74 years
- Age at death due to dementia = A_AD+T_DAD= 85+3=88 years
- Age at death due to other causes = Reference age (70) + T_DOC = 70+6= 76 years

Given these values and consulting the schematic diagram (Figure 2.-), we could state that:

- At the age of 60 for instance this individual was in the preclinical phase according to epidemiological classification and Thal phase 2 according to pathological classification.
- The individual would die of other causes at 76 years in the preclinical stage and Thal phase 3 before entering the stage of dementia.

Allocation of individuals and incidence and prevalence measurement

The computation of the entities that pass through each of the phases/stages allowed both the annual incidence and prevalence to be ascertained. We used two variables for this purpose.

SYSTEM PARAMETERS

In the following, we provide a brief description of the main system parameters.

Input population

The focus of the study was the population over 40 years of age. As mentioned above, to avoid a long warming-up period all cohorts were introduced at the same time, assigning them an appropriate health status at that point. This was not, however, sufficient, since the incidence was also analyzed. Therefore, the entry of entities to the system was staggered, with the whole population over 40 years of age being entered the first year (year 2009) effectively 'filling' the system, and from 2010 (year of analysis) onwards only the incident cohort of individuals of 40 years of age being entered each year.

Population data were obtained from the Spanish National Institute of Statistics (INE) [4]. An empirical distribution was used to determine the age at entry of each individual.

Time to death due to other causes

Individuals with AD are at the same time exposed to other risks such as unrelated diseases, traffic accidents, etc. Therefore, some may die before the disease developed. We assumed that individuals in the preclinical and prodromal stages had general population mortality rates. On the other hand, once that they enter the stage of dementia it was assumed that they could die either due to the disease or other causes.

The DES makes it necessary to consider time explicitly. Time to an event was modeled by using the Gompertz distribution, which as a function of age, can be expressed as follows [5]

$\text{Time to an event} = \frac{1}{\beta} \times \ln(1 - \frac{\beta}{\alpha} \times \ln(1 - \mathrm{u}) \times \mathrm{e}^{-\beta \times \mathrm{Age}}$ (1).

This equation includes a uniformly distributed random factor between 0 and 1 (u) and two parameters α and β which define the characteristics of the distribution. These parameters, α and β, are estimated from a linear regression of a logarithmic transformation of the rates of occurrence of the event with respect to age, expressed in Equation 2

$$\ln(\text{Rate}) = \beta_0 + \beta_1 \times \text{Age} \quad (2)$$

where

$$\beta = \beta_1 \quad (3)$$

$$\alpha = e^{\beta_0} \quad (4).$$

Time to dementia

Time to dementia was the time between the reference age and the loss of capacity to perform instrumental activities of daily living. It was also the time to be taken as reference for other calculations since dementia is the only known stage of AD. For calculating the time until dementia we took a reference age of 50 years. AD is rarely expressed clinically before the age of 60. However, almost 1% of the population between 60 and 65 years suffer it. Therefore, the reference age had to be set somewhat earlier so that there could be incident cases of AD-dementia by that age. In order to build a Gompertz function, AD-dementia incidence rates given by Fratiglioni were used [6] with the same methodology as previously described [5].

Time from the appearance of Aβ deposits to AD-dementia

Currently, it is not possible to measure the Aβ level in living individuals, rather it can only be ascertained by post-mortem brain biopsy, so the duration of this stage is unobservable. However, Braak et al described the prevalence of Thal phases in terms of prevalence by age group [1]. This information allowed us to ascertain this time by calibration, prevalence in general terms being the consequence of the interaction of incidence and duration, and calibration involving the fitting of these two in order to obtain the observed prevalence.

The first step was achieved when the model reproduced the incidence and prevalence of the dementia stage. The second step consisted in estimating the duration of the preclinical stage by adjusting the model until the results matched the pattern observed for Thal phase 1 in the Braak et al study [1].As the parameter Thal phase 1 prevalence couldn't be directly observed, calibration was performed using a random search method [7]. Calibration is the process of determining the values of unobservable parameters by constraining model output to replicate observed data [7]. The epidemiological parameters for Thal phases 2 and 3 were also calculated by applying the same method.

This same procedure was used to determine the beginning of each of the Thal phases.

Time from MCI to AD-dementia

Considering that the rate of conversion is constant over time and applying the exponential function (mean time=1/rate), we obtained the duration of prodromal stage.

Survival in the clinical stage

These values were obtained from Dodge's clinical series [8].

In order to obtain stochastic values, an exponential function was built. The mean value was obtained from a polynomial function (Equation 5), the parameters for which are listed in Table 1.

$$\lambda = \text{Median survival in clinical stage} = \text{b0} + \text{b1} \times \text{Age} + \text{b2} \times \text{Age}^2 \qquad (5)$$

Statistical assessment of validation and calibration

Calibration was performed using a random search method [7]. For each phase, we established the time from the beginning of that phase to dementia. Thal phase's prevalence was obtained in 2010 and grouped by age and sex. The model was assessed using the following goodness-of-fit statistics: the correlation coefficient (R), normalized mean square error (NMSE), fractional bias (FB), fractional variance (FV) and the fraction of predictions within a factor of two (FAC2) [7].The same approach was applied to assess the validation process for dementia incidence and prevalence.

MODEL RESULTS

In the following, we describe the results in detail.

Validation results

On the one hand, the dementia stage is the one for which results have been validated, since it is the only stage for which data are available, specifically the following: 1) the incidence rate, and 2) the prevalence rate. The incidence rates obtained were compared with the results of the meta-analysis of Fratiglioni et al [6] (Figure 3 and Table 2) and the prevalence with the one held values reported by Lobo et al [9] (Figure 4 and Table 3). Note that incidence is not an input but the result of the interaction between the competing risks of having dementia and death due to other causes. Likewise, the prevalence is not an input; rather it is determined by dementia incidence and dementia survival time.

On the other hand, Thal phases have been validated. For this, we compared the prevalence results obtained by Braak et al [1] and those obtained with the model. This demonstrated that the calibration had been performed correctly (Figure 5 and Table 4).

Goodness-of-fit results

All the statistics used to assess the goodness-of-fit were within the established criteria as shown in Tables 5 and 6.

REFERENCES

1. Braak H, Thal DR, Ghebremedhin E, Del Tredici K. Stages of the pathologic process in Alzheimer disease: age categories from 1 to 100 years. J Neuropathol Exp Neurol. 2011 Nov;70(11):960-9.
2. Knapp M, Prince M. Dementia UK: the full report. Alzheimer's Society. London. 2007.
3. Karnon J, Stahl J, Brennan A, Caro JJ, Mar J, Möller J; ISPOR-SMDM Modeling Good Research Practices Task Force. Modeling using discrete event simulation: a report of the ISPOR-SMDM Modeling Good Research Practices Task Force--4. Value Health. 2012;15:821-7.
4. Instituto Nacional de estadística www.ine.es. Visited 25/11/2013
5. Roman R, Comas M, Hoffmeister L, Castells X. Determining the lifetime density function using a continuous approach. J Epidemiol Community Health 2007; 61: 923–25.

6. Fratiglioni L, Launer LJ et al. Incidence of dementia and major subtypes in Europe: A collaborative study of population-based cohorts. Neurology 2000;54(11 Suppl 5); S10-5.
7. Stout NK, Knudsen AB, Kong CY, McMahon PM, Gazelle GS. Calibration methods used in cancer simulation models and suggested reporting guidelines. Pharmacoeconomics. 2009;27:533-45.
8. Dodge HH, Shen C et al. Functional transitions and active life expectancy associated with Alzheimer disease. Arch Neurol 2003; 60:253-259.
9. Lobo A, Launer LJ et al. Prevalence of dementia and major subtypes in Europe: A collaborative study of population-based cohorts. Nurologic Diseases in the Elderly Research group. Neurology 2000;54 (11 Suppl 5):S4-9.
10. Jicha GA, Carr SA. Conceptual evolution in Alzheimer's disease: implications for understanding the clinical phenotype of progressive neurodegenerative disease. J Alzheimers Dis. 2010; 19:253-72.
11. Petersen RC, Roberts RO, Knopman DS, Boeve BF, Geda YE, Ivnik RJ, Smith GE, Jack CR Jr. Mild cognitive impairment: ten years later. Arch Neurol. 2009 Dec;66(12):1447-55.

Figure 1.- Conceptual model of the representation of AD natural history

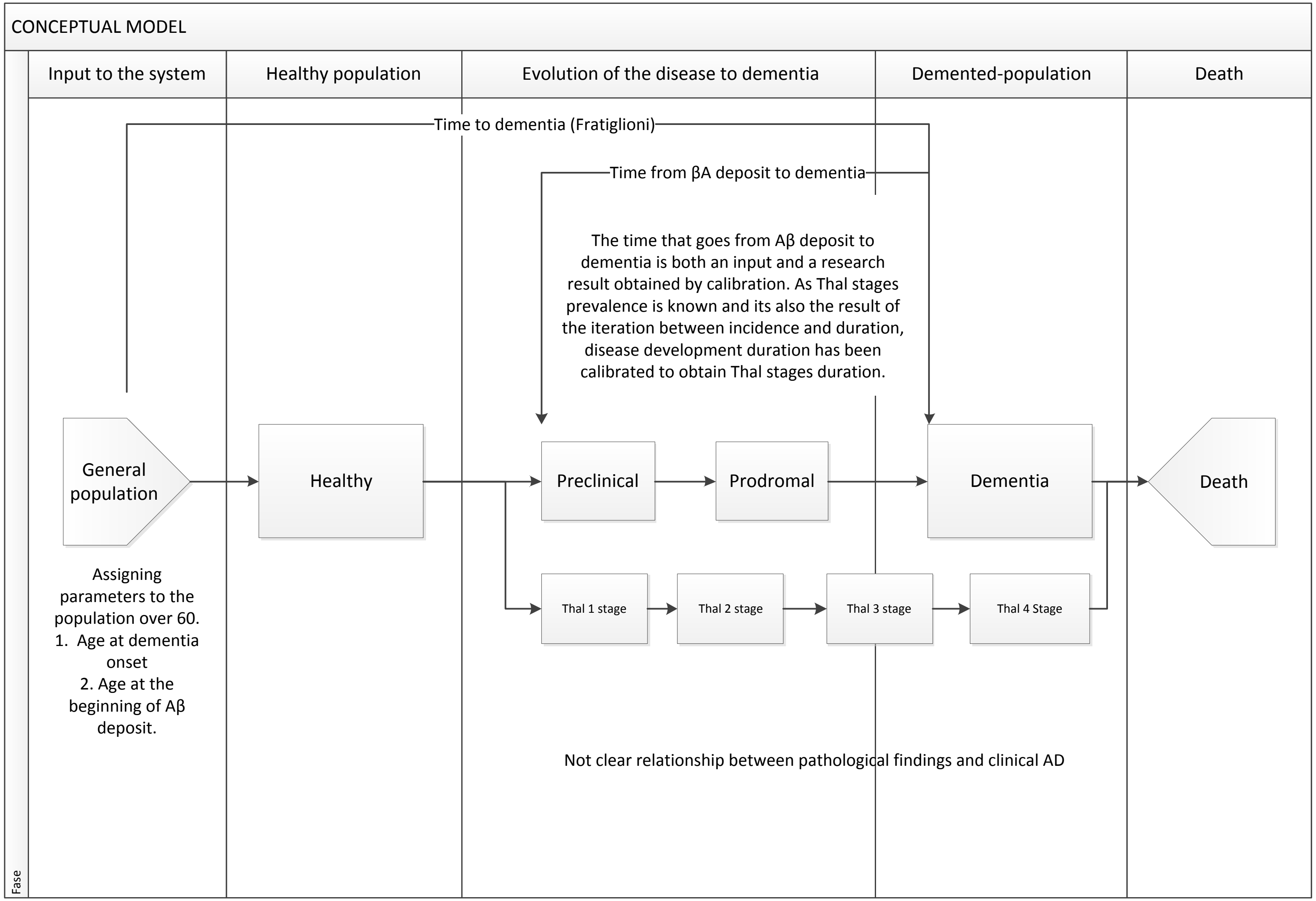

Figure 2.- Example of assigning times to an entity.

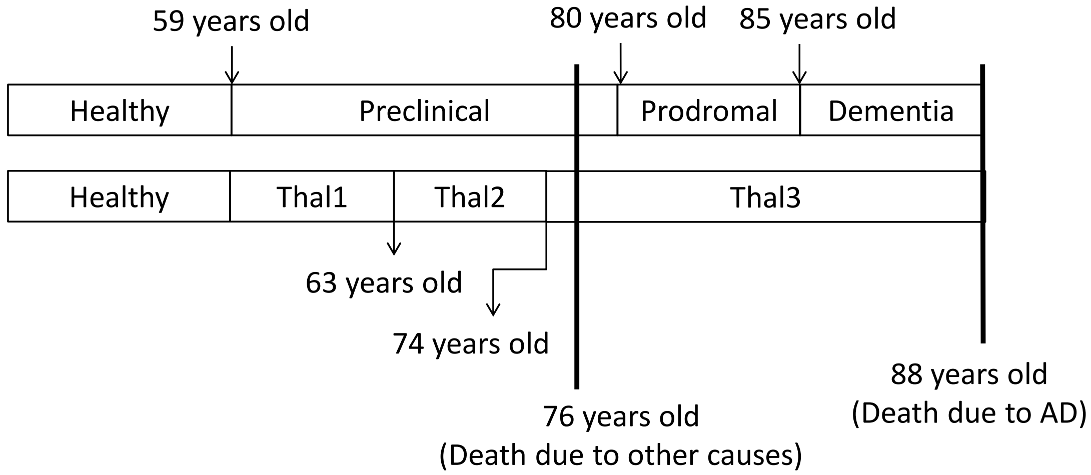

Figure 3.- Validation of the incidence per thousand

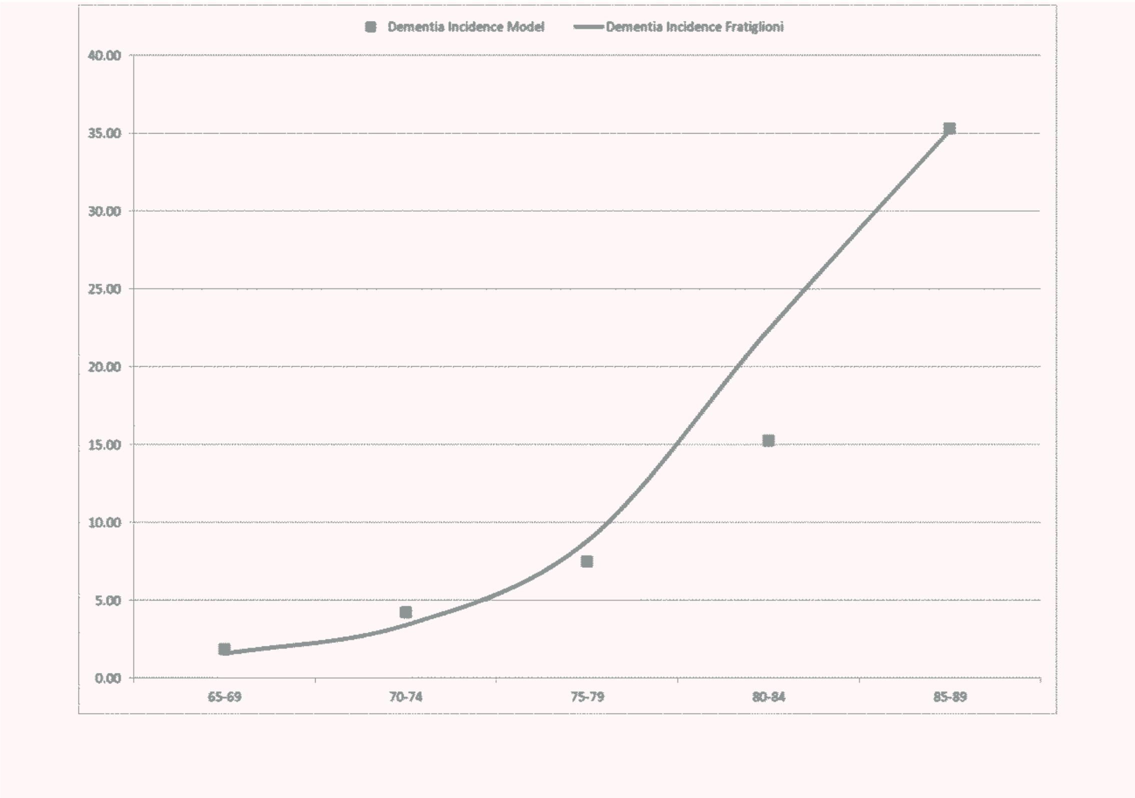

Figure 4.- Prevalence validation

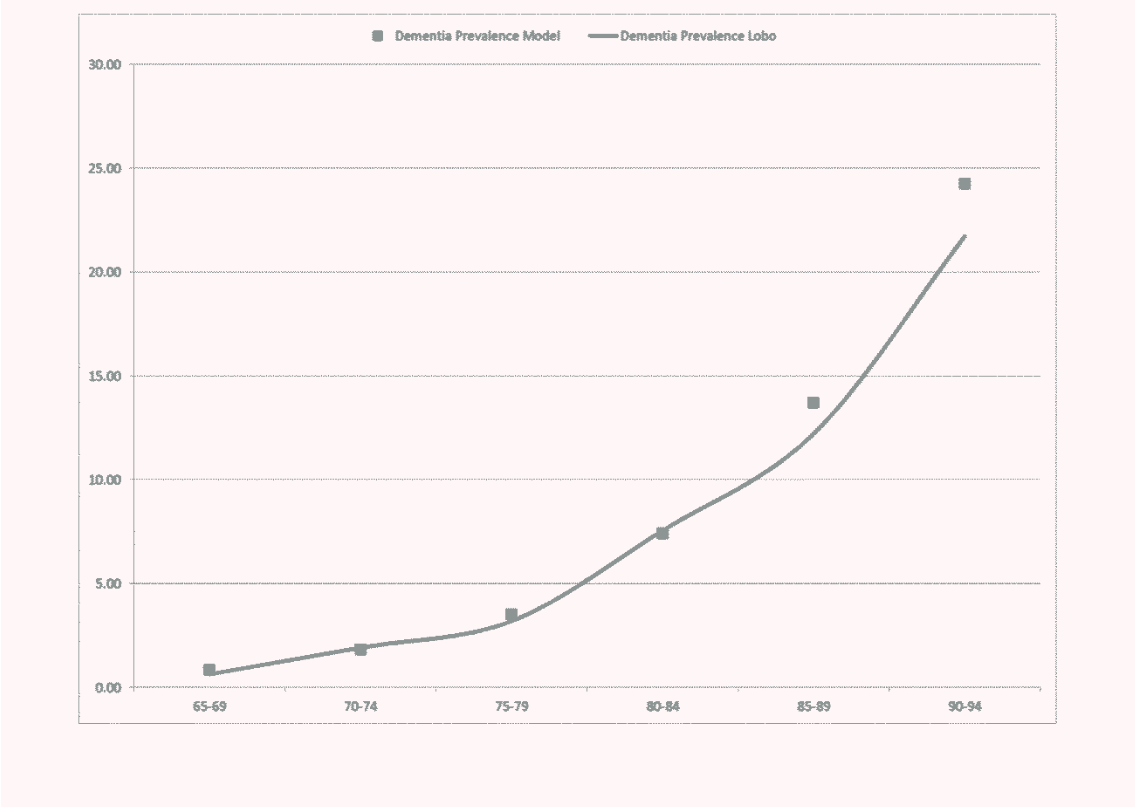

Figure 5.- Thal phases validation

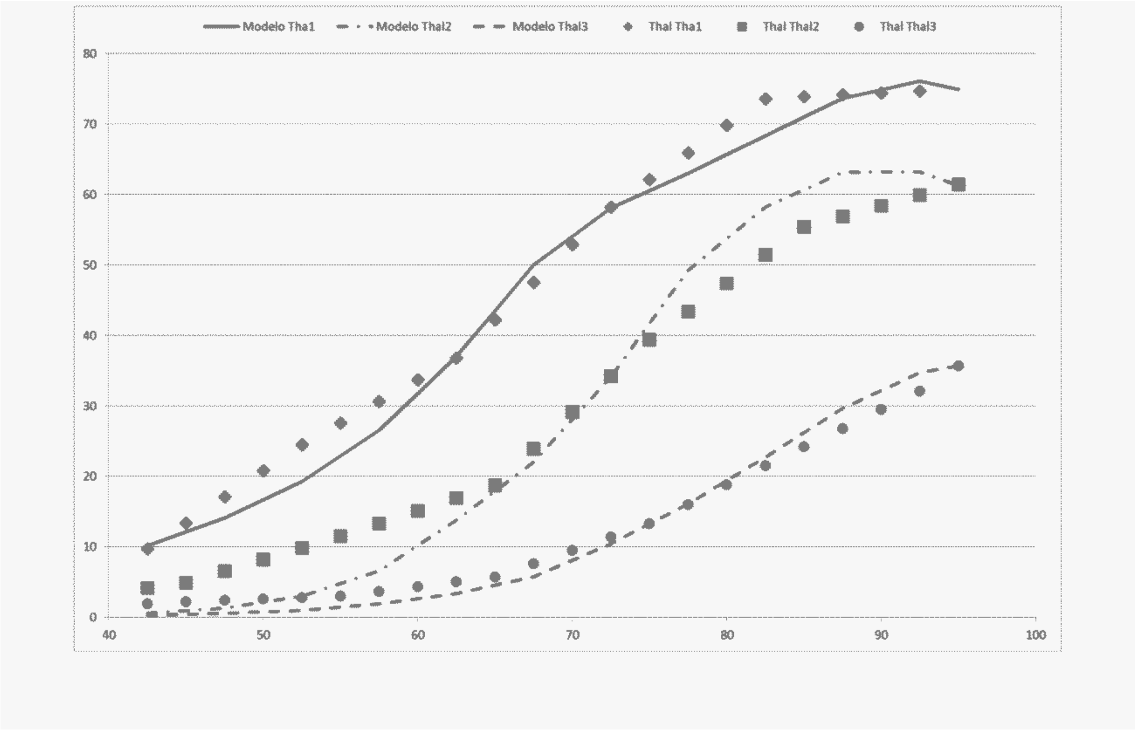

Table 1.- Parameters of the model

	Source	Function	Parameter	Male	Female
Time to death due to other causes	[4]	Gompertz	ln(α)	-10.022	-11.922
			β	0.090	0.108
Time to AD-dementia	[6]	Gompertz	ln(α)	-17.825	16.72
			β	0.164	0.157
Clinical stage survival	[8]	Polynomial	b0	30.835	42.767
			b1	-0.447	-0.709
			b2	0.002	0.003
Time in prodromal stage	[10,11]	Exponential	λ	5	5

Table 2.- Validation of the incidence per thousand

	Dementia incidence/1000	
Age group	Model	Fratiglioni et al
60-64	0.7	-
65-69	1.9	1.6
70-74	4.2	3.4
75-79	7.5	8.8
80-84	15.3	22.4
85-89	35.3	35.2
90-94	0.7	

Table 3.- Prevalence validation

	Dementia prevalence (%)	
Age group	Model	Lobo et al
60-64	0.4	
65-69	0.9	0.7
70-74	1.8	1.9
75-79	3.5	3.2
80-84	7.4	7.6
85-89	13.7	12.2
90-94	24.3	21.7

Table 4.- Thal stages validation

	Thal phases prevalence (%)					
	Model			Braak et al		
Age	Thal1	Thal2	Thal3	Thal1	Thal2	Thal3
40-45	10.2	0.6	0.3	8.5	4.2	1.9
45-50	14.1	1.3	0.6	13.4	6.6	2.4
50-55	19.3	3.0	1.0	20.8	9.9	2.8
55-60	26.6	6.6	1.9	27.6	13.3	3.7
60-65	37.0	13.8	3.4	33.7	16.9	5.0
65-70	50.0	22.0	5.8	42.2	23.9	7.6
70-75	58.1	34.2	10.5	52.9	34.2	11.4
75-80	63.0	49.2	16.1	62.1	43.4	16.0
80-85	68.4	58.2	22.8	69.8	51.4	21.5
85-90	73.7	63.2	29.7	73.9	56.9	26.8
90-95	76.1	63.2	34.7	74.4	59.9	32.1

Table 5.- Goodness of fit for validation (dementia incidence and prevalence)

	Criteria	Incidence	Prevalence
Correlation coefficient (R)	(> 0·8)	0.78	0.84
Normalized mean squared error (NMSE)	(< 0·5)	0.06	0.03
Fractional bias (FB)	[-0·5, 0·5]	0.11	-0.09
Fractional variance (FV)	[-0·5, 0·5]	0.23	0.14
Factor of two	(> 0·8)	1.00	1.00

Table 6.- Goodness of fit for calibration of Thal phases incidence and prevalence

	Criteria	Thal 1	Thal 2	Thal 3
Correlation coefficient (R)	(> 0·8)	0.95	0.95	0.95
Normalized mean squared error (NMSE)	(< 0·5)	0.00	0.02	0.02
Fractional bias (FB)	[-0·5, 0·5]	-0.03	0.01	0.03
Fractional variance (FV)	[-0·5, 0·5]	0.12	0.21	0.14
Factor of two	(> 0·8)	1.00	1.00	1.00
